# Salutatory

**Published:** 1873-11

**Authors:** 


### SALUTATORY.

Our readers will note with regret that the old flag-editorial, bearing honored names, long and favorably known to the Profession of the South and of the whole country, has been lowered from the mast-head, and a new and unfamiliar pennon now floats in its stead.

Of our predecessors, so long identified with this JOURNAL: impaired in health by continual professional labor, chafed by much wearing of the harness, longing for a little quiet and rest; it would ill-become us to speak in terms of fulsome eulogy, which could add nothing to their well earned fame, and but offend their modest worth.

For ourself and our Associates we have nothing to say. We have no apologies to make; we have no pledges to offer. With an earnest desire to maintain courteous and amicable relations with our brethren of the medical quill, and feeling in our hearts an entire willingness to labor diligently in defense of the RIGHT, we mount the tripod.

ROBERT BATTEY, M.D.

### BUSINESS NOTICE.

It will be seen that the Editorial and Business Management of the Journal has passed into new hands; Dr. W. F. Westmoreland still retaining his proprietorship of the Journal.

For the future all correspondence for either the Editorial or Business Department should be addressed to

ROBERT BATTEY, M.D.,
Editor and Business Manager.

---

We have received from Dr. J. M. Toner, of Washington, D. C., advance sheets of his new work, now in press, entitled "Dictionary of Elevations of the United States." The forthcoming of Dr. Toner's work will be looked for with interest by many of our readers engaged in the study of the natural history of disease. Such students will find in Dr. Toner's "Dictionary" a source of information bearing upon the subject not easily obtainable elsewhere. We take the liberty of extracting the following statistics, viz:

| | GEORGIA. | NEW YORK. | MASS. |
|---|---|---|---|
| Average altitude above the sea | 575 | 800 | 400 |
| Per cent. deaths to population, 1850 | 1.10 | 1.47 | 1.95 |
| Per cent. deaths to population, 1860 | 1.21 | 1.21 | 1.73 |
| Per cent. deaths to population, 1870 | 1.15 | 1.58 | 1.77 |
| Per cent. of deaths from consumption to total mortality in 1860 | 3.83 | 17.63 | 22.74 |
| Per cent. of deaths from consumption to total mortality in 1870 | 6.43 | 16.77 | 19.93 |
| By diseases of the nervous system, 1870 | 11.16 | 13.96 | 11.93 |
| By diseases of the respiratory system, 1870 | 16.51 | 12.22 | 10.80 |

We have been often impressed with the fact that there has been a very marked increase in the prevalence and mortality of consumption under our own observation, at Rome, Ga., within the past twenty-five years. This ratio of increase we may roughly estimate at, perhaps, *ten to one*.

Rome is situated near the center of the Cherokee purchase, or Cherokee country, and was formerly the seat of council of the nation or tribe. Since the removal westward of the Indians, in 1838, the region, on account of its attractive climate and fertile soil, has been rapidly filled up with white population—and a small proportion of negroes.

At first, the absence of railroad communication, the low price of lands, the fertile soil, the abundance of game, and the laxity of the administration of law, determined the settlement of this region with a rough, hardy, robust population, possessing but little of this world's goods; unaccustomed to the enervating luxuries of higher social life; living and rearing their families in rude log cabins, roofed with roughly split clap-boards, through which the summer's breeze and the winter's blast found easy way. The daily life of the pioneers was of the simplest and plainest character; their diet generous and wholesome, but free of all effort to tempt a flagging appetite—to them almost unknown. Hardy woodsmen, huntsmen, farmers, and land-speculators, they lived in the open air and were active and vigorous in habit.

Here we have the conditions which give the greatest immunity from pulmonary consumption; and our own recollection, extending back to 1847, is, distinctly, that the disease, as existing in the region referred to, was, to us, almost unknown.

In process of time, the country became permeated by railroads; the population increased; the products of the soil augmented in value; cotton culture was introduced; the primitive forest fell in homage to the progress of agriculture. The value of lands steadily increased until the pioneers had become enriched by the advance, and feeling the need of a broader domain for their enlarged families of hardy boys, as well as an instinctive longing for wider and better hunting grounds, they disappeared, one by one, rifle in hand, with their moving trains of stock and scanty household plunder, towards the setting sun. By reason of the great advance in lands, the purchasers were men of greater means, and, therefore, of more culture and accustomed to more of the (so-called) comforts and luxuries of life. They were, for the most part, slave-holders, and in general led a (physically) more indolent and more self-indulgent life.

Coincidently with this change in population—and, we have thought, in consequence of the change—the prevalence of consumption has greatly and steadily increased. Indeed, we have heretofore believed that the greater per centage of the disease was attributable alone to the change in population, coupled with the enervating effects of luxury and ease and more sedentary employments.

In the tables of Dr. Toner, however, we think we clearly see another cause now operating to similar result. It will be observed in our tabular extract from his Dictionary, that there has been a very notable increase in the relative mortality from consumption in Georgia during the decade, (1860 to 1870), an increase of nearly one hundred per cent. This increase we do not think can be satisfactorily accounted for by the facts we have stated.

If we observe the tables a little more closely, we find that there has been no such increase in Florida—the difference being comparatively trivial; that there has been a slight *decrease* in Minnesota; whilst the increase in Louisiana is approximately as 10 to 7; in Texas, as 10 to 7; in Alabama as 10 to 6½; in Mississippi, as 10 to 6; in North Carolina, as 10 to 5; in South Carolina, as 10 to 4½. Upon the other hand, we find that there has been an equally notable *decrease* in the ratio of deaths from consumption in the Middle States and New England: e.g., in New York, the ratio of decrease is about as 10 to 10½; Massachusetts, as 10 to 11½; Connecticut, as 10 to 11½ plus.

It is well known that previous to our late war, the customary resorts for consumptives from the Northeastern States, in search of health, were, for the most part, Florida and Minnesota; while other States of the South have come more into notice, in this particular, since the war. South Carolina, especially, has urged her claims as a health resort, of late years. We must consider, also, the influence which the abolition of slavery, and other changes incident to the political situation, has probably exerted in rendering these States more attractive to invalids from New England.

Since the war, too, there has been an unwonted influx of permanent population from the more Northern States. With this new population comes, of course, its hereditary and acquired predispositions. But when these influences are duly weighed, and full allowance made for them, we are of opinion that there yet remains an appreciable increase in the number of cases occurring in families long established in the locality.

Our cursory examination of Dr. Toner's tables suggests sundry other topics of thought and comparison which we may return to at some future time.

---

We have received from the Surgeon-General's office at Washington a copy of the annual report of that officer to the Secretary of War.

We notice, in comparison of statistics of sick and wounded among the white and colored troops of the army, that while the ratio of sick per thousand of mean strength among the whites was 1,963, and among the blacks 1,708, the proportion of *deaths* from all causes is reversed—being 1 to 118 among whites, and 1 to 81 among the blacks.

The recorded surgical cases have reached the large figure, 259,907,—a large mass of information for future utilization. The specimens added to the Army Medical Museum during the year number little short of one thousand; and the importance and value of this magnificent collection is well attested by the statement of the Surgeon-General: "During the year, twenty-two thousand five hundred and sixty visitors registered their names."

It is to be hoped that the suggestion of the Surgeon-General, that the edition issued of the Medical and Surgical History of the War is insufficient, coupled with the very high estimate of its value, expressed and felt by the entire medical profession of the country, may have influence with Congress in the ordering an additional issue. We are pleased to learn that the second part is now passing through the press.

---

We have been favored with a copy of the "Memorial of the American Medical Association with Regard to the Rank of the Medical Corps of the United States Army."

Our brethren of the army present their grievances in a forcible light, and as they have received the support of the Surgeon-General, and the hearty co-operation of the American Medical Association, we trust that their prayer will be granted at the present Congressional session.

In this connection, we observe that the Surgeon-General states, in his report, that there are now sixty-four vacancies in the medical corps, and remarks: "The inducements of pay and rank, as at present established, are not sufficient to make the service attractive or remunerative to physicians already engaged in practice."

---

Puerperal Septicæmia—Treatment.—Dr. T. Morton, Surgeon to Kilburn Dispensary, says: "I make it my rule never to repress diarrhœa; when there is improvement without it, to leave well alone; when there is no improvement without it, to lose no time in setting it up."—*Obstet. Jour. Great Brit. and Ireland.*

